# Iron oxide nanoparticles coated with Glucose and conjugated with Safranal (Fe_3_O_4_@Glu-Safranal NPs) inducing apoptosis in liver cancer cell line (HepG2)

**DOI:** 10.1186/s13065-024-01142-1

**Published:** 2024-02-15

**Authors:** Somayeh Mikaeili Ghezeljeh, Ali Salehzadeh, Somayeh Ataei-e Jaliseh

**Affiliations:** Department of Biology, Rasht Branch, Islamic Azad University, Rasht, Iran

**Keywords:** Apoptosis, Drug delivery, Liver cancer, Fe_3_O_4_@Glu-Safranal, Safranal

## Abstract

Magnetic nanoparticles can be considered a reliable tool for targeted drug delivery to cancer tissues. Based on this, in this study, the anticancer effect of iron oxide nanoparticles coated with glucose and conjugated with Safranal (Fe_3_O_4_@Glu-Safranal NPs) on a liver cancer cell line (HepG2) was investigated. Physicochemical properties of nanoparticles were characterized using FT-IR, XRD, VSM, EDS-mapping, SEM and TEM imaging, zeta potential, and DLS analyses. MTT test was used to investigate the inhibitory effect of nanoparticles on cancer and normal cell lines. Also, the reactive oxygen species (ROS) level, the population of apoptotic cells, and cell cycle analysis were evaluated in control and nanoparticle-treated cells. The synthesized particles were spherical, in a size range of 17–49 nm, without impurities, with a surface charge of − 13 mV and hydrodynamic size of 129 nm, and with magnetic saturation of 22.5 emu/g. The 50% inhibitory concentration (IC_50_) of Safranal, Fe_3_O_4_, Fe_3_O_4_@Glu-Safranal and Cisplatin drug on liver cancer cells were 474, 1546, 305 and 135 µg/mL, respectively. While, the IC_50_ of Fe_3_O_4_@Glu-Safranal for normal cell line was 680 µg/mL. Treating liver cancer cells with nanoparticles significantly increased the population of apoptotic cells from 2.5% to 34.7%. Furthermore, the population of the cells arrested at the G2/M phase increased in nanoparticle-treated cells. Due to the biocompatibility of the constituent compounds of these nanoparticles, their magnetic properties, and their inhibitory effects on cancer cells, Fe_3_O_4_@Glu-Safranal NPs can be further considered as a promising anticancer compound.

## Introduction

Cancer is considered one of the most common non-infectious diseases and the most important cause of human mortality. In recent years, the rate of cancer incidence and mortality has been increasing in many parts of the world, so this disease is considered one of the most important threats to human health. Liver cancer is one of the most common and deadly types of cancer. The annual rate of morbidity and mortality of this disease is estimated 905,000 and 830,000 cases, respectively, which has caused serious concerns in the field of human health [1]. Although surgery and removal of tumor tissue is considered the primary treatment option, effective treatment of this disease, especially in its advanced stages, is largely based on chemotherapy methods. However, chemotherapy drugs also have limitations due to insufficient effectiveness, unwanted side effects, and inability to treat all types of liver tumors, especially metastatic types [2].

As a novel approach in anticancer chemotherapy, directing medicinal compounds to tumor sites can increase the effectiveness and reduce the side effects of medicinal compounds. In this method, drug compounds are conjugated with a carrier and directed to the target tissues. As a result, only the target cells are affected by the drug and other cells are spared from the toxic effects of the drug [3]. The use of magnetic nanoparticles can be considered a reliable tool for targeted drug delivery to cancer cells. Furthermore, feasibility of magnetic field assisted control of magnetic nanoparticles behavior has proven them suitable candidates for targeted drug delivery [4]. Due to their good magnetic ability, suitable biocompatibility, and acceptable stability, the use of iron oxide nanoparticles in targeted drug delivery has attracted the attention of many researchers [3–5]. In this method, medicinal compounds are conjugated to iron nanoparticles and the particles are directed to the target cancer tissues using a magnetic field. In addition, by coating these particles with appropriate materials, in addition to increasing their biocompatibility, it is possible to improve the drug's internalization into cancer cells, where it can exert its anticancer effects [5, 6].

Safranal is the most abundant chemical in saffron essential oil and the main compound affecting saffron characteristic aroma. Safranal has several biomedical properties, including antioxidation, protective effect against drug-induced damage, and cytotoxic effect against several cancer cells [7]. The anticancer activity of safranal on various cancer cell lines has been reported [8]. The underlying anticancer mechanism of safranal has been proposed through inhibition of microtubule polymerization, causing DNA damage and inhibiting DNA repair mechanisms, as well as inhibiting DNA replication and transcription [9, 10]. Moreover, it was found that safranal could enhance the transcription of proapoptotic genes, which leads cancer cells into apoptosis. Therefore, Safranal could be considered an efficient anticancer compound that could be employed in cancer chemotherapy.

As described above, Iron oxide NPs have been widely studied for their potential use in selective targeting of cancer cells. However, the addition of Glucose, as a functionalizing agent, and Safranal, as an anticancer compound, to Iron oxide NPs and their potential use in anticancer chemotherapy has not been investigated. This combination may provide a foundation for further research in the field of formulation of novel, safe, and more effective anticancer compounds. Due to the anticancer effect of safranal on various cancer cells, and the magnetic properties of Iron oxide nanoparticles (NPs), in this work, Iron oxide NPs were synthesized, coated with Glucose, and conjugated with Safranal. Finally, the anticancer effect of Fe_3_O_4_@Glu-Safranal NPs on a human liver cancer cell line was investigated.

## Materials and methods

### Reagents and materials

All chemicals used were of analytical grade and were used as received without any further purification. The Safranal was purchased from Sigma-Aldrich company with CAS number: 116-26-7.

### ***Synthesis of Fe***_***3***_***O***_***4***_***@Glu-Safranal NPs***

At first, Fe_3_O_4_ NPs were synthesized as follows: 7.57 g of FeCl_3_.6H_2_O and 3.17 g of FeCl_2_.4H_2_O were suspended in distilled water. The mixture was heated at 80 °C for 1 h, and then, 40 mL of concentrated NH_3_ solution was added and the heating continued for another hour. Next, the mixture was centrifuged and Fe_3_O_4_ NPs were collected, washed, and dried at 70 °C for 8 h [11].

In the next step, Fe_3_O_4_ NPs were coated with glucose as follows: one gram of Fe_3_O_4_ NPs and 0.5 g of d-glucose were suspended in deionized water, the mixture was sonicated for 30 min, and then, the mixture was heated at 180 °C for 3 h. Finally, the particles were collected by centrifugation at 6000 rpm, washed, and dried at 60 °C for 5 h.

To conjugate Fe_3_O_4_@Glu with Safranal, one gram of Fe_3_O_4_@Glu and 0.1 g of Safranal were suspended in deionized water and shaken for 24 h. Next, Fe_3_O_4_@Glu-Safranal NPs were harvested, washed, and freeze-dried (steps are shown in the following formula).

$$\begin{aligned} & 5.75\,\text{g Fe{Cl}}_3\cdot 6\text{H}_2\text{O}+3.17\,\text{g\,Fe{Cl}}_2\cdot4\text{H}_2\text{O}+300\,\text{mL\,d{H}}_2\text{O}\rightarrow\text{Fe}_3\text{O}_4 \, \text{NPs}\\ & 1\, \text{g {Fe}}_3\text{O}_4\,\text{NPs}+0.5\,\text{g D-Glucose}(\text{C}_6\text{H}_{12}\text{O}_6)\rightarrow \text{Fe}_3\text{O}_4@ \text{Glu NPs} \\ & 1\, \text{g {Fe}}_3\text{O}_4@ \text{Glu NPs}+0.1 \, \text{g Safranal}(\text{C}_{17}\text{H}_{26}\text{O}_4)\rightarrow \text{Fe}_3\text{O}_4@ \text{Glu-Safranal NPs}\end{aligned}$$

### Physicochemical properties of particles

Fourier Transform Infrared (FT-IR) assay was used to characterize the functional groups of the particles. The assay was performed using a Nicolet IR-100 FT-IR device in a range of 500–4000 cm^−1^. X-ray Diffraction (XRD) assay was used to evaluate the physical phase and crystal structure of Fe_3_O_4_@Glu-Safranal NPs (Co-Ka X-radiation, k = 1.79 Å). Elemental mapping (EDS-mapping) of the Fe_3_O_4_@Glu-Safranal NPs was done to evaluate the elemental composition of the particles (TESCAN Mira3). Furthermore, the size and morphology of the particles were characterized by SEM (TESCAN Mira3) and TEM (Zeiss EM-900) imaging. The surface charge and hydrodynamic size of the particles were analyzed by a zeta-sizer instrument (Malvern Instruments Ltd, 6.32). Magnetic properties of the Fe_3_O_4_@Glu-Safranal NPs were also analyzed using a VSM analysis (LBKFB magnetometer, Daghigh Kavir Kashan, Iran).

### Antiproliferative property of Fe_3_O_4_@Glu-Safranal NPs

The antiproliferative effect of Fe_3_O_4_@Glu-Safranal on HepG2 (a liver cancer cell line) and HEK293 (a normal human cell line) cells was investigated by MTT assay. Cell culture was performed in Dulbecco’s modified Eagle medium (DMEM) medium. After preparation of the cell monolayer in 96-well cell culture plates, different quantities of the NPs that were already dispersed in distilled water were added to the wells, so that, the final exposure concentrations of 0–1000 µg/mL were provided. In addition, the antiproliferative effect of Safranal on HepG2 cells was investigated by treating cell monolayers with different concentrations of safranal. After overnight incubation at 37 °C, the medium was aspirated, and 200 µl of the MTT (2-(4,5-dimethythiazol-2-yl)-2,5-diphenyltetrazolium bromide) solution was added. The plates were further incubated for 4 h, and next, the content of the wells was replaced with 200 µl of DMSO. The plates were stored at room temperature for 30 min and the OD_590_ of the wells was measured by a Bio-Rad microplate reader. Finally, the 50% inhibitory concentration (IC_50_) of Fe_3_O_4_@Glu-Safranal for the HepG2 and HEK293 cells was calculated as follows [12, 13]:

$$Inhibition (\mathrm{\%})=\frac{Abs \,of \,control-Abs \,of \,Test}{Abs \,of \,control}\times 100$$

### Reactive oxygen species (ROS) level

The ROS level generated due to the exposure to Fe_3_O_4_@Glu-Safranal NPs in HepG2 cells was measured as follows: At first, a monolayer of the cells was grown in 6-well plates (3.0 × 10^5^ cells/well). Next, the NPs (at their 50% inhibitory concentration) were added to the cells and incubated for 24 h and at 37 °C. After incubation, the cells were collected, washed with PBS, and treated with 2′,7′-dichlorodihydrofluorescein (H2DCF). After incubation in the dark at ambient temperature for 1 h, the cells were washed and fluorescence intensity was measured [14].

### Cell apoptosis/necrosis

Flow cytometry assay was used to investigate the effect of exposure to Fe_3_O_4_@Glu-Safranal NPs on the population of apoptotic and necrotic cells in the liver cancer cell line. For this purpose, HepG2 cells were treated with a 50% inhibitory concentration of nanoparticles for 24 h and then, the cells were stained by propidium iodide and Annexin V (Roche, Germany). Untreated cells were also considered as the negative control. Finally, the population of apoptotic and necrotic cells was quantified by the Partec™ flow cytometry instrument (Germany).

### Cell cycle analysis

Flow cytometry analysis was also used to determine the cell cycle phases in Fe_3_O_4_@Glu-Safranal NPs cancer cells and control cells. At first, HepG2 cells were prepared in 6-well plates, and treated with the nanoparticles, as described above. After incubation for 24 h, the cells were collected, washed, and fixed with cold ethanol. Next, the cells were stained with propidium iodide and treated with RNase A (100 µg/mL). Cell cycle phases were determined by a flow cytometry analysis based on the quantity of cell DNA content.

### Statistical analysis

Statistical analysis was performed by one-way analysis of variance (ANOVA) using SPSS, 16.0 software. P-values of less than 0.05 were considered statistically significant.

## Results

### Physicochemical properties of nanoparticles

**Fig. 1:**
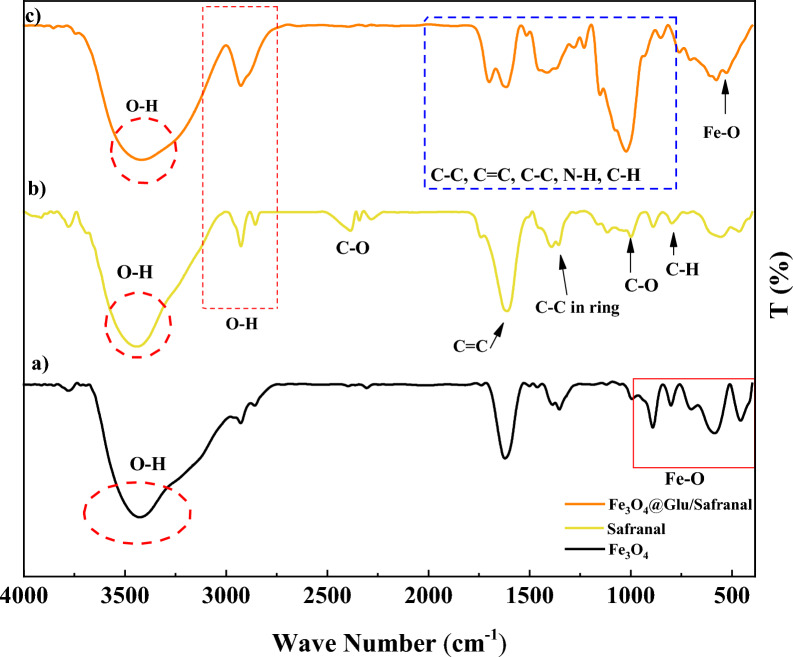
FT-IR spectrogram of Fe_3_O_4_@Glu-Safranal, Safranal, and Fe_3_O_4_ nanoparticles

FT-IR assay was used to evaluate the functional groups on the particles (Fig. 1 and Table 1). According to the results, in the FT-IR spectrum for Fe_3_O_4_ NPs, intense absorption peaks at 420, 580, and 624 cm^−1^ are respectively associated with the Fe–O bonds related to Fe^2+^ and Fe^3+^ ions located in octahedral sites and Fe^3+^ ions located in tetrahedral sites. This finding suggests the formation of the Fe_3_O_4_ structure. Since the peaks related to maghemite (γ-Fe_2_O_3_) are in the wavelength range of 580–1650 cm^−1^, the presence of a peak at 420 cm^−1^ indicates the purity of the formed magnetic phase.

**Table 1:** Presentation of functional groups in Fe_3_O_4_@Glu-Safranal NP

Band	Fe_3_O_4_	Safranal	Fe_3_O_4_@Glu-Safranal
	Wave number (cm^−1^)		
Fe–O	599	–	570
O–H	3452	2931–3488	2932–3468
C–H	–	798	852
C–O	–	1005–2392	1032–2380
C–C	–	1391	1393
N–H	–	–	1602

In the FT-IR spectrum related to Safranal, the peaks at 803, 100, and 1393 cm^−1^ correspond to C–H, C–O, and C–C bonds, respectively. Also, the absorption peaks at 1612 and 2423 cm^−1^ are related to C=C and C–O bonds, respectively. Furthermore, the peaks at 2935 and 3406 cm^−1^ are related to OH bonds. Considering the presence of the peaks related to both materials in the structure of the composite, it can be concluded that the composite has been correctly synthesized.

**Fig. 2:**
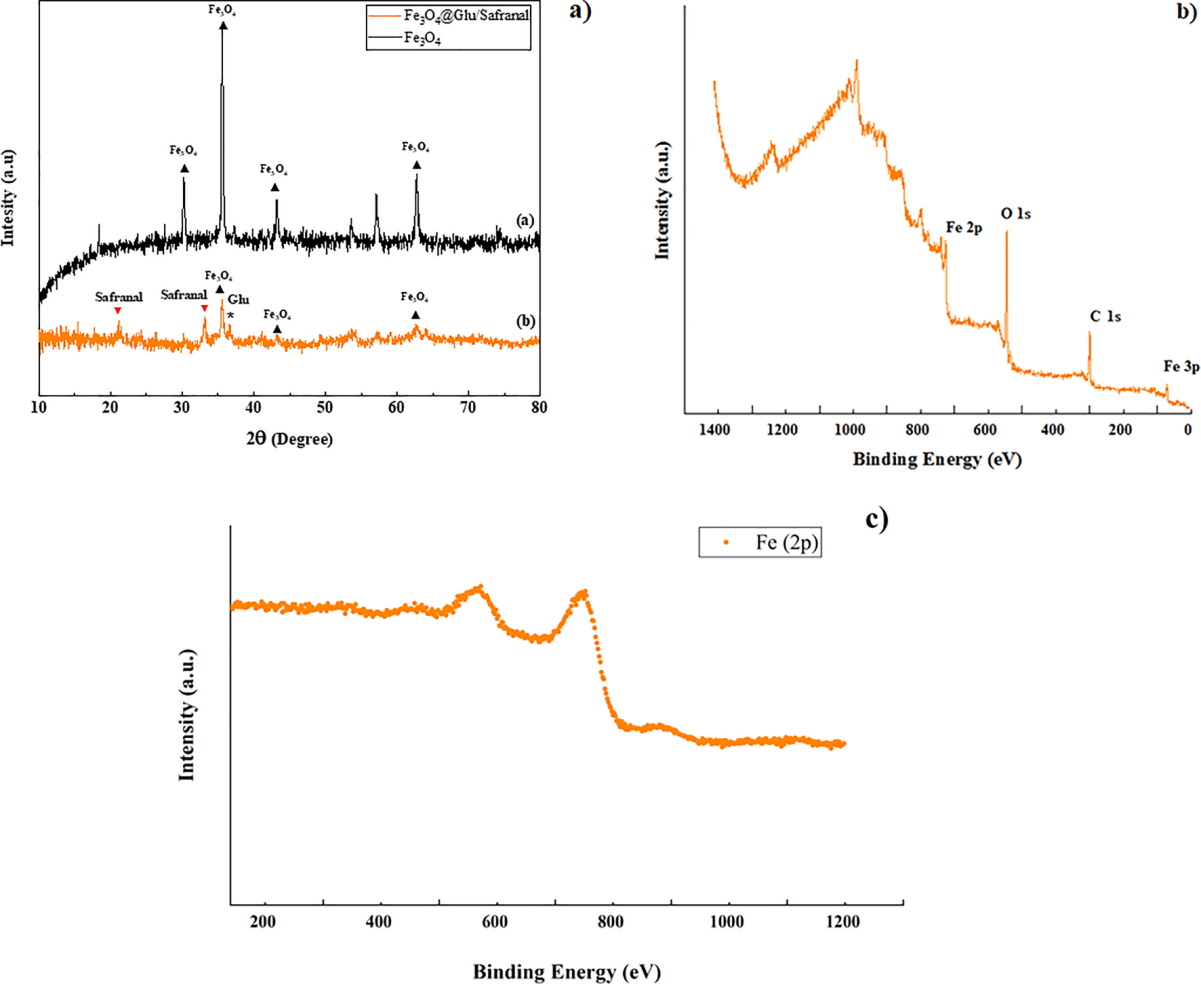
**a** XRD and **b** XPS analyses of Fe_3_O_4_@Glu-Safranal nanoparticles. **c** Fe 2p spectrum

According to the XRD assay (Fig. 2a), the values of 2θ equal to 30, 35, 57 and 63 degrees are related to iron oxide nanoparticles, which is in accordance with card number 0863–03 [15]. In spectrum b, the 2θ value of 36.62 degrees seems to be related to glucose, and the 2θ values of 21.15, and 31.56 degrees are associated with the presence of Safranal, and the 2θ values of 35.5, 42.7, and 62.52 degrees are also related to iron oxide [16].

To determine the chemical compositions and electronic structure of Fe_3_O_4_@Glu-Safranal, X-ray photoelectron spectroscopy (XPS) measurements was performed and a typical full XPS spectrum was shown in Fig. 2b. The spectrum indicated the presence of carbon, oxygen, and iron. Because the atomic sensitivity factor of Fe is much higher than those of C and O, the negligible and weak peaks of Fe imply that the Fe_3_O_4_ was uniformly and continuously coated by Glu-Safranal. For the Fe 2p spectrum (Fig. 2c), two peaks at 701.8 and 742.3 eV correspond to Fe 2p_3/2_ and Fe 2p_1/2_ of Fe_3_O_4_, respectively [17, 18]. The spin–orbit splitted Fe 2p peaks are broad due to a small chemical shift between Fe^2+^ and Fe^3+^ presented in Fe_3_O_4_ [19]. Moreover, the absence of shakeup satellite peak situated at ~ 719 eV, which is the fingerprint of the electronic structures of Fe_2_O_3_ [20], also confirms the Fe_3_O_4_ species rather than Fe_2_O_3_. This is an important character to distinguish Fe_3_O_4_ (magnetite) and γ-Fe_2_O_3_ (maghemite) since their same crystalline structure but different valence state of iron ions.

**Fig. 3:**
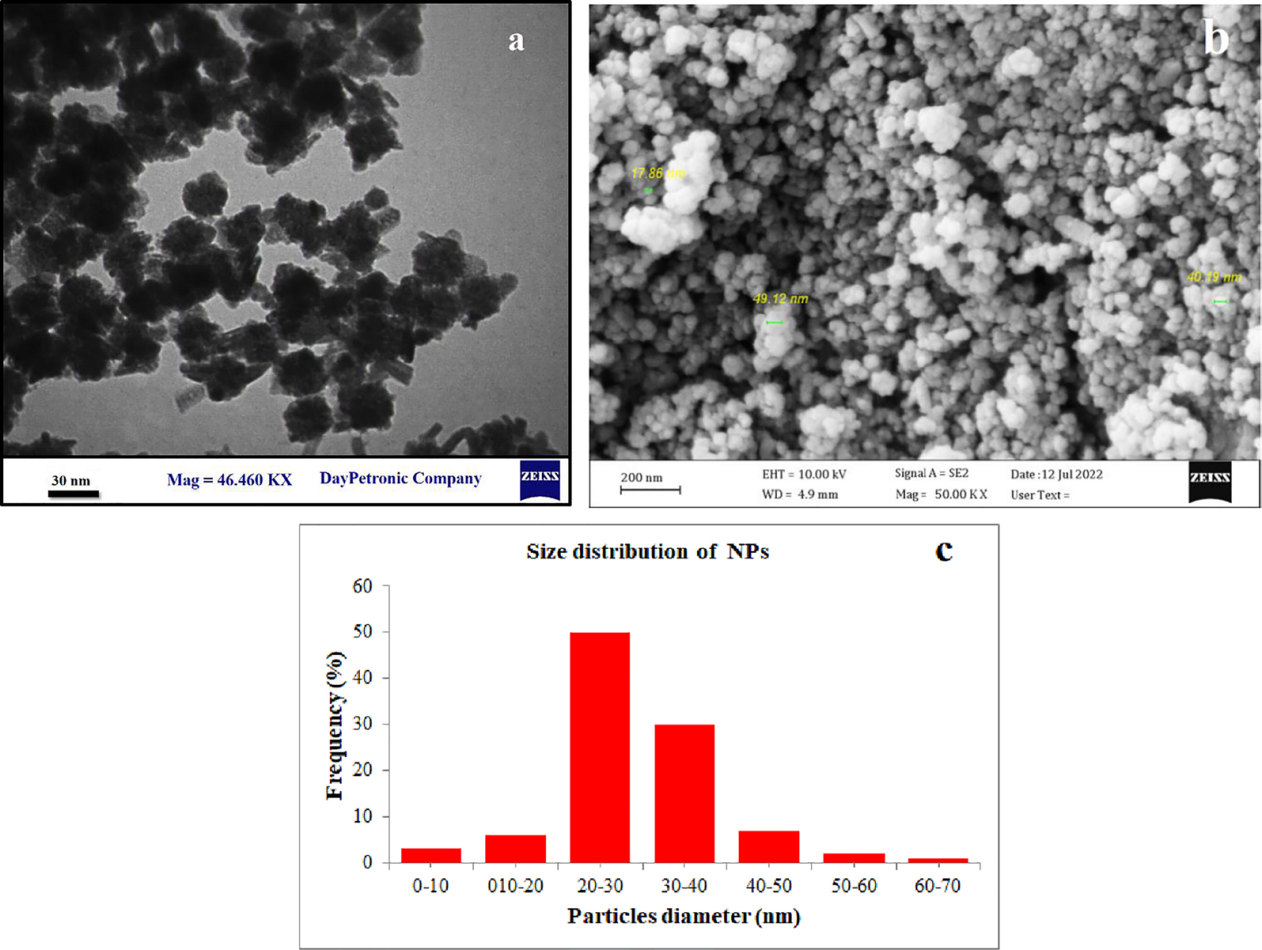
**a** TEM, **b** SEM and **c** particle size distribution of Fe_3_O_4_@Glu-Safranal nanoparticles. The synthesized particles were spherical and in a size range of 17–49 nm

**Fig. 4:**
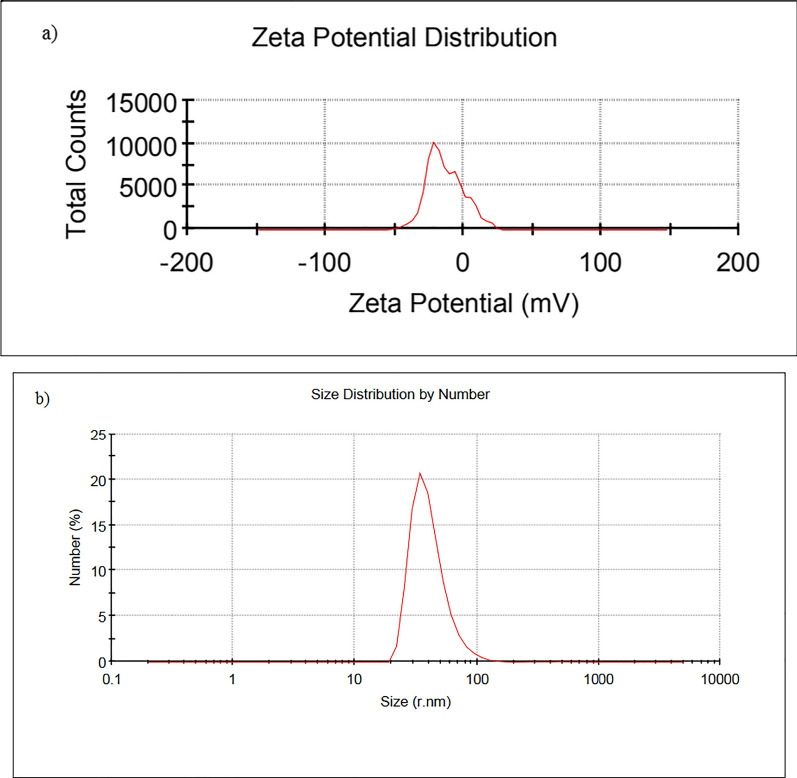
**a** Zeta potential, and **b** DLS analysis of Fe_3_O_4_@Glu-Safranal nanoparticles. Surface charge of the nanoparticles was − 13 mV and hydrodynamic size was 129 nm

According to electron microscopy imaging, the synthesized particles were spherical and synthesized in a size range of 17–49 nm. Figure 3 displays the SEM and TEM images of the synthesized particles. Moreover, the surface charge and hydrodynamic size of the particles were − 13 mV and 129 nm, respectively which indicate the proper stability and size of the particles in an aquatic environment (Fig. 4).

**Fig. 5:**
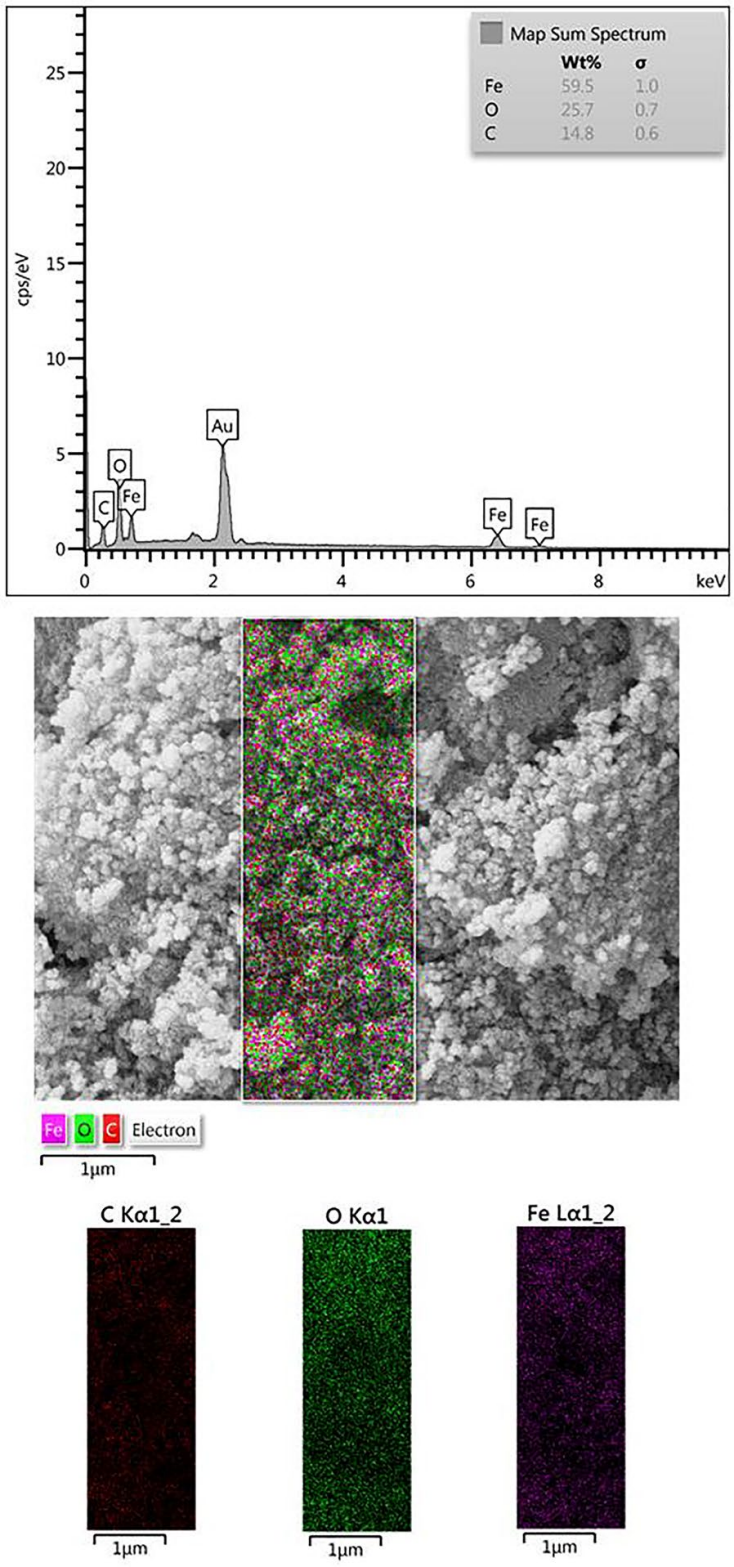
Elemental mapping of Fe_3_O_4_@Glu-Safranal nanoparticles. The particles contained C, Fe, and O atoms, and no elemental impurity was observed

**Fig. 6:**
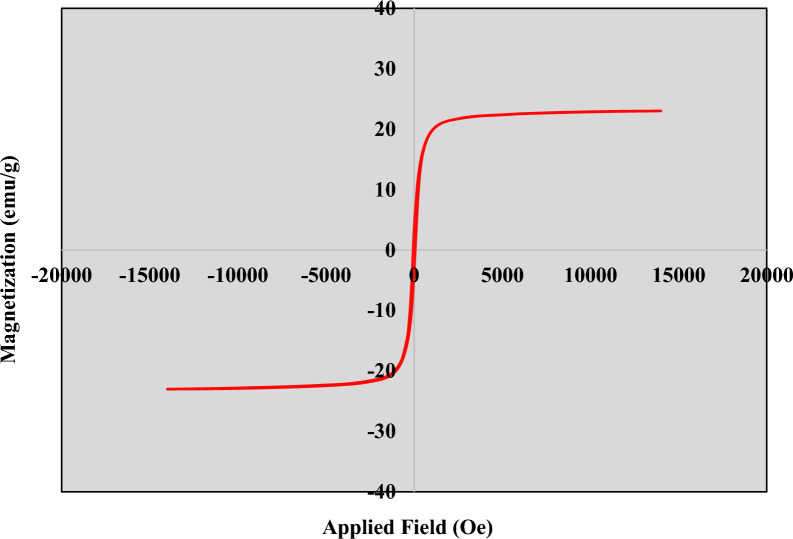
Magnetic saturation curve of Fe_3_O_4_@Glu-Safranal nanoparticles. The particles had magnetic properties and the maximum magnetic property was 22.5 emu/g which was observed at 6000Oe

Elemental mapping of the particles revealed that Fe_3_O_4_@Glu-Safranal NPs contained Fe, O, and C atoms that indicates the synthesized particles had no elemental impurity. Figure 5 shows the elemental mapping of the Fe_3_O_4_@Glu-Safranal NPs. According to the VSM analysis, the synthesized particles had magnetic properties and the maximum magnetic property was 22.5 emu/g which was observed at 6000Oe (Fig. 6).

### MTT assay

**Fig. 7:**
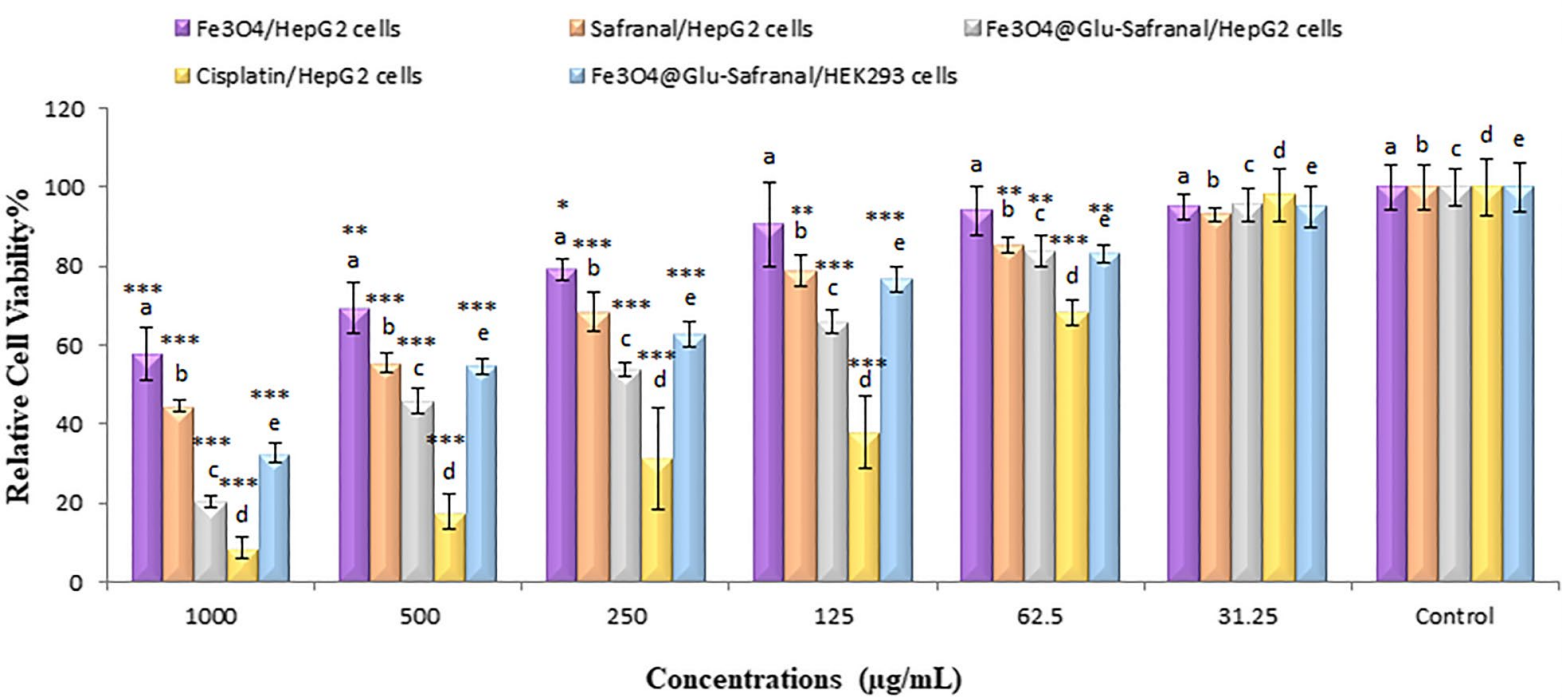
MTT assay showed that the IC_50_ of Safranal, Fe_3_O_4_, Fe_3_O_4_@Glu-Safranal and Cisplatin on liver cancer cells were 474, 1546, 305 and 135 µg/mL, respectively. The IC_50_ of Fe_3_O_4_@Glu-Safranal on normal cell line was 680 µg/mL

MTT assay was used to determine the antiproliferative effect of Safranal, Fe_3_O_4_ NPs, Fe_3_O_4_@Glu-Safranal NPs and Cisplatin drug on cancer cell line. Also, the antiproliferative effect of Fe_3_O_4_@Glu-Safranal NPs on normal cell line was evaluated. According to the results, at concentrations of ≥ 62.5 µg/mL, the viability of cancer cells was significantly reduced by these materials. The IC_50_ of Safranal, Fe_3_O_4_, Fe_3_O_4_@Glu-Safranal and Cisplatin drug on liver cancer cells were 474, 1546, 305 and 135 µg/mL, respectively. Meanwhile, the IC_50_ of Fe_3_O_4_@Glu-Safranal on normal cell line was 680 µg/mL. The results were presented in Fig. 7.

### ROS level

**Fig. 8:**
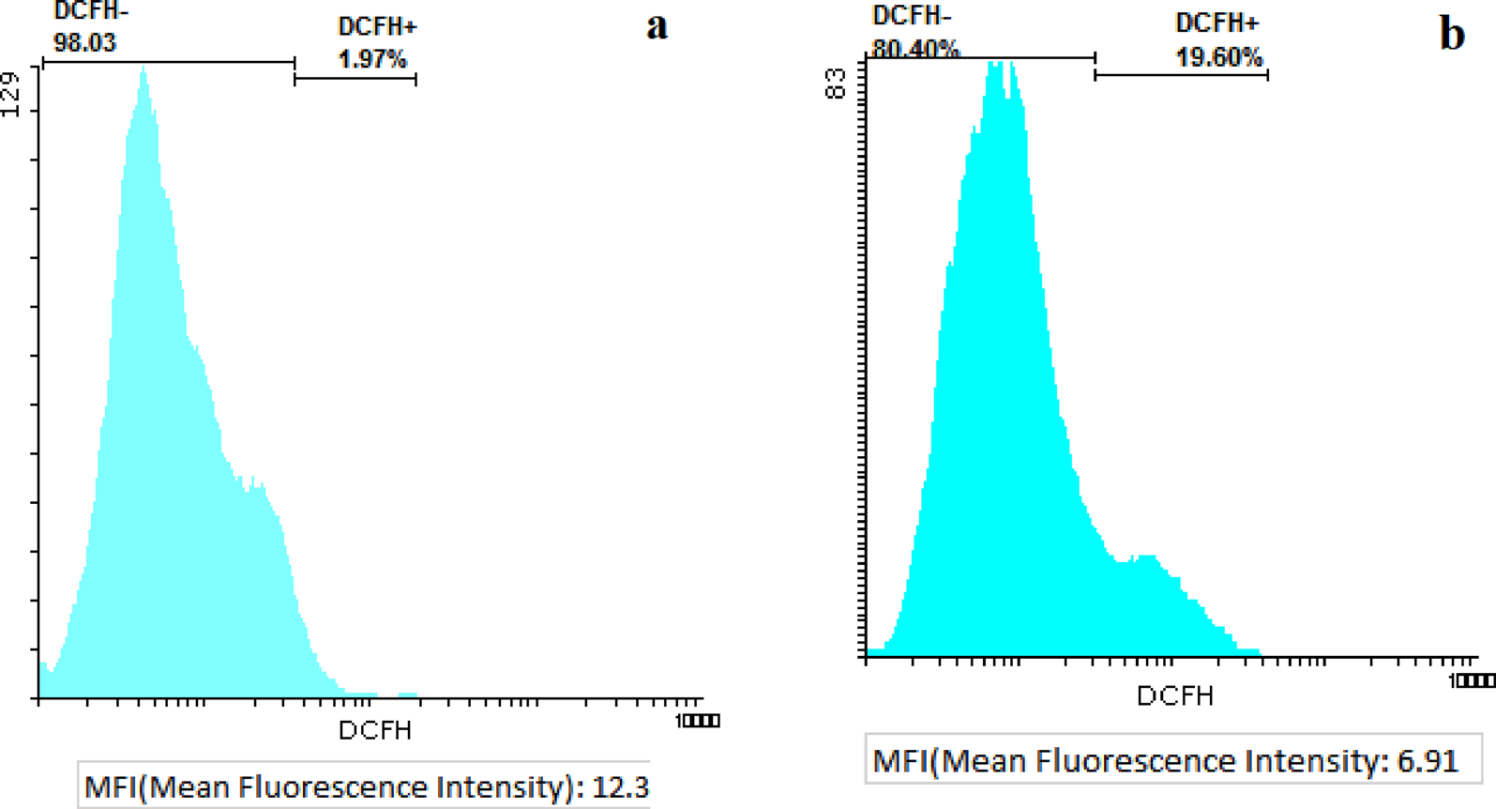
ROS generation in **a** control and **b** Fe_3_O_4_@Glu-Safranal treated cells. Treating cancer cells with the nanoparticles resulted in a considerably higher ROS generation

To evaluate the effect of oxidative stress on the cytotoxic mechanism of Fe_3_O_4_@Glu-Safranal NPs, the ROS level was quantified in nanoparticle-treated and control cells. According to the results, the DCFH + level in control cells was 1.97%, while in nanoparticle-treated cells increased to 19.60%, which suggests the generation of ROS molecules following treatment of cancer cells with the nanoparticles. Figure 8 shows the ROS levels in nanoparticles treated and control cells.

### Cell apoptosis/necrosis

**Fig. 9:**
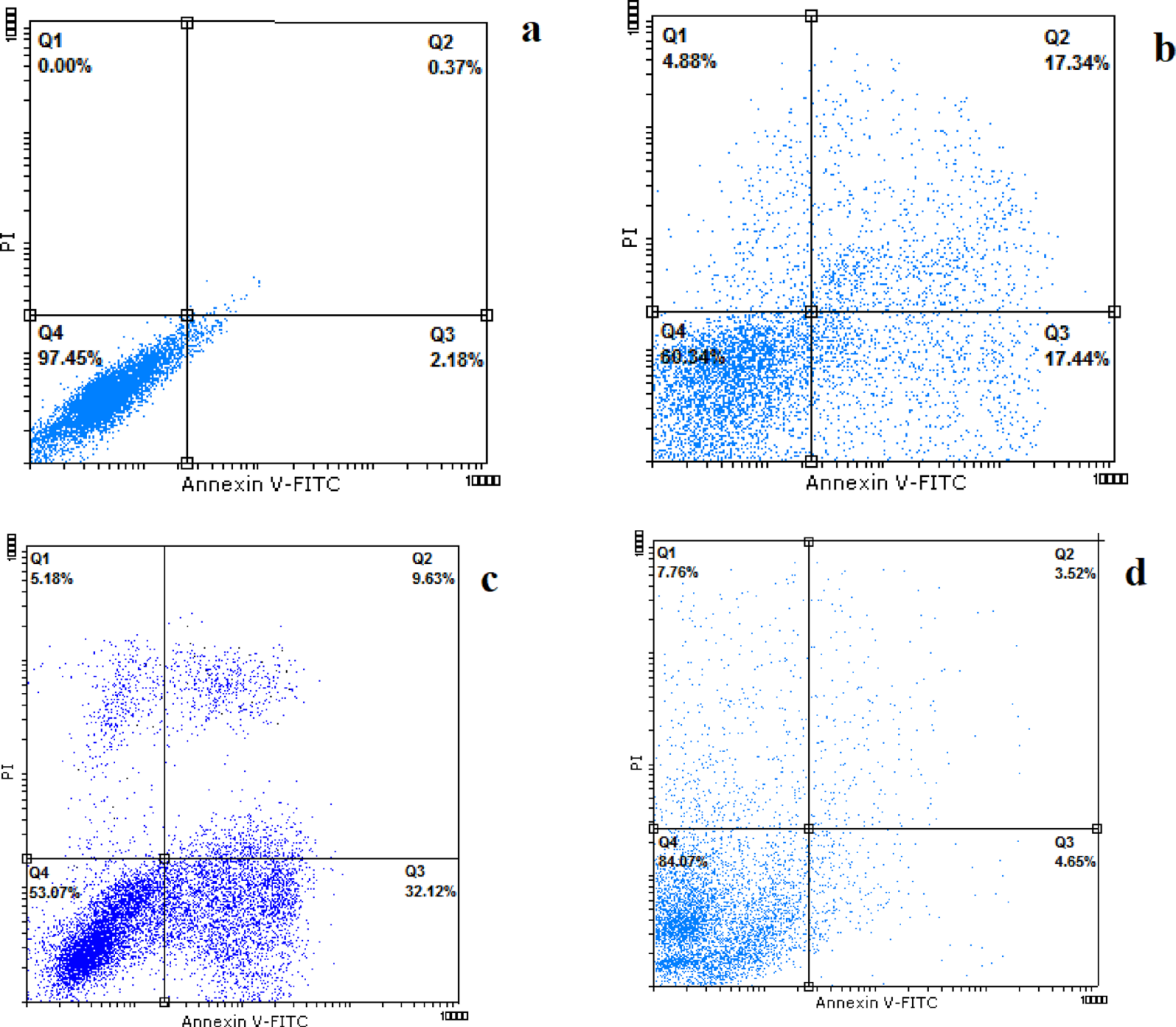
Flow cytometry analysis **a** control, **b** Fe_3_O_4_@Glu-Safranal NPs, **c** Cisplatin drug and **d** Fe_3_O_4_ NPs treated cancer cells. Treating with the Fe_3_O_4_@Glu-Safranal NPs and Cisplatin drug considerably increased the population of apoptotic cells. Q1: necrotic cells, Q2: late apoptosis, Q3: primary apoptosis, and Q4: live cells

Flow cytometry analysis was performed to determine the frequency of apoptotic; necrotic and healthy cells in nanoparticles treated, and control cancer cells. We found that the frequency of cell necrosis, primary, and late apoptosis in control cells were 0.00, 2.18, and 0.37%, respectively. In contrast, treating HepG2 cells with Fe_3_O_4_@Glu-Safranal NPs increased the population of cell necrosis, primary, and late apoptosis to 4.88, 17.44, and 17.34%. Meanwhile, treating cancer cells with Cisplatin drug increased the population of cells in necrosis, primary and late apoptosis condition to 5.18, 32.12, and 9.63%, respectively. Also, treating cancer cells with Fe_3_O_4_ increased the population of cells in necrosis, primary and late apoptosis condition to 7.76, 4.65, and 3.52%, respectively, which were lower than Fe_3_O_4_@Glu-Safranal NPs and Cisplatin drug. The results were presented in Fig. 9.

### Cell cycle analysis

**Fig. 10:**
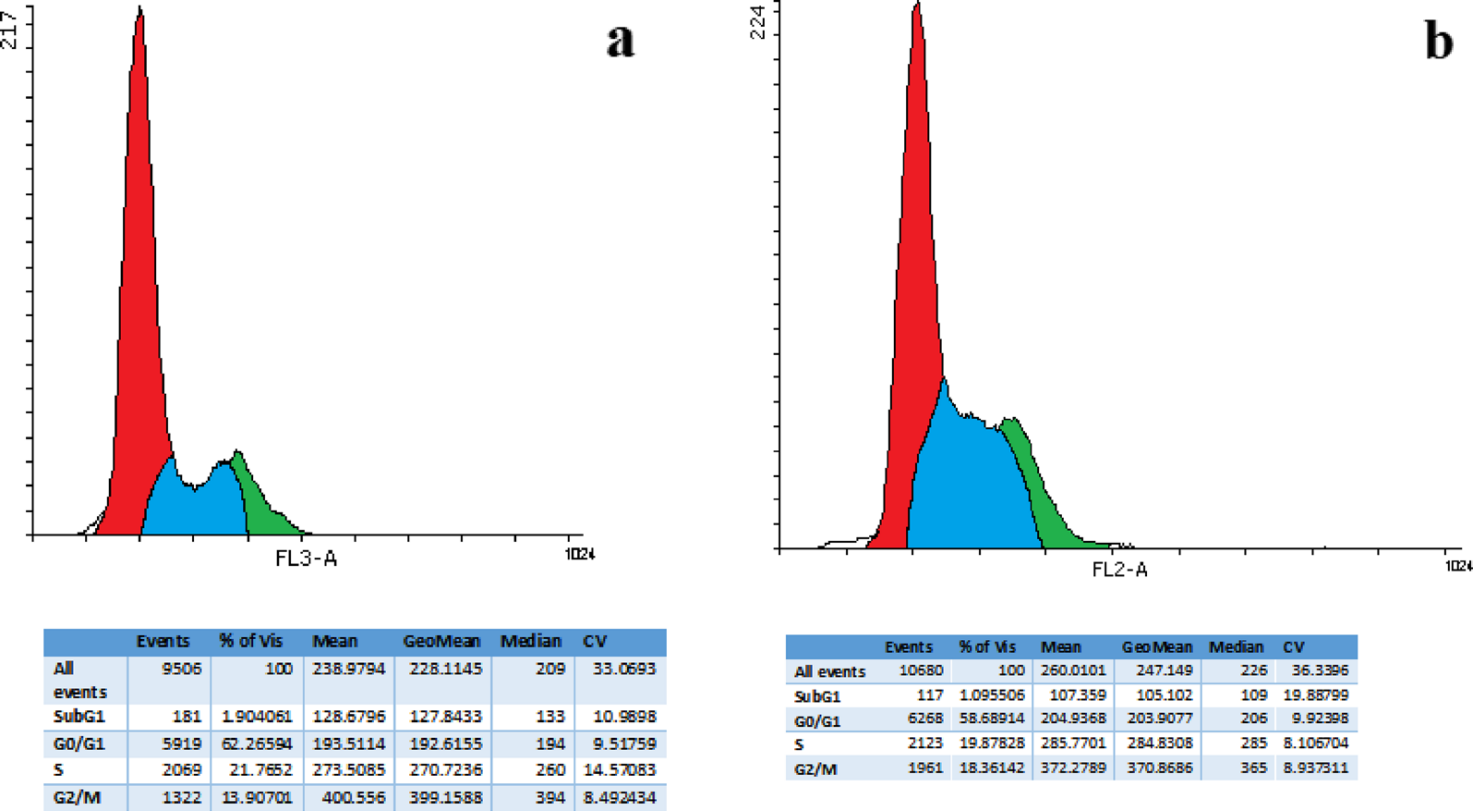
Cell cycle analysis of **a** control and **b** Fe_3_O_4_@Glu-Safranal treated cells showed an increased population of the cells arrested in G2/M Phase

Cell cycle analysis of control and nanoparticle-treated cells showed that in the control group, 1.9, 62.2, 21.7, and 13.9% of cells were at the sub-G1, G0/G1, S, and G2/M phases, respectively. The frequency of the cells at the sub-G1, G0/G1, S, and G2/M phases in treated cells was 1.09, 58.6, 19.8, and 18.3%, respectively. The results indicated an increase in the population of the cells at the G2/M phase following treatment with Fe_3_O_4_@Glu-Safranal NPs, while the population of the cells of other phases decreased. Figure 10 presents the cell cycle analysis of the control and treated cells.

## Discussion

The increase in the rate of liver cancer mortality on the one hand and the insufficient efficiency of current treatment methods on the other hand, show the need to obtain new and more efficient drugs for the effective treatment of liver cancer. The clinical use of most drug candidates for anticancer chemotherapy faces limitations, the most important of which is lack of biocompatibility and required efficacy to achieve effective treatment, as well as considerable unwanted side effects [21].

Although replacing the use of natural compounds instead of chemically synthesized molecules can be a solution to improve biocompatibility and reduce the side effects of anticancer drugs, these compounds generally lack sufficient therapeutic effects. The use of targeted drug delivery approaches can improve the effectiveness and reduce unwanted side effects of medicinal compounds. Therefore, in this research, the anticancer effect and mechanism of Fe_3_O_4_@Glu-Safranal NPs on a gastric cancer cell line were investigated.

Physicochemical characterization of the synthesized NPs revealed that the Fe_3_O_4_@Glu-Safranal NPs were synthesized in a nanoscale size range, had a proper agglomeration level, and had no elemental impurity. DLS is an effective tool to characterize dynamic parameters of nanoparticles including the diffusion coefficient and particle size within a colloidal system. The polydispersity Index (PDI Index) in DLS analysis was equal to 0.536. This value shows the NPs are mono dispersed. According to literature related to colloid stability, values of zeta potential equal to ± 10–20 mV are relatively stable. In this study this value was − 13 mV that shows the Fe_3_O_4_@Glu-Safranal NP is relatively stable [22]. In addition, due to the observed magnetic properties, the synthesized particles could be used for directed delivery using an external magnetic force. The efficiency of magnetic nanoparticles for directed drug delivery in in-vivo and in-vitro models has been reported [23–25].

Investigating the antiproliferative effect of Fe_3_O_4_@Glu-Safranal NPs on live cancer and normal fibroblast cells indicated that the nanoparticles had a significantly stronger inhibitory effect on cancer cells than normal human cells. In addition, Fe_3_O_4_@Glu-Safranal NPs had a much stronger toxic effect than Safranal against cancer cells, which can be concluded by comparing the IC_50_ value of each compound. The antiproliferative effect of Fe_3_O_4_@Glu-Safranal NPs seems to be mainly related to the Safranal. Previous studies reported the cytotoxic effect of Safranal.

It was found that Safranal can inhibit polymerization of microtubules which leads to inhibition of cell proliferation. Naghshineh et al. reported that microtubule polymerization decreased significantly in the presence of safranal, regardless of its concentration. Furthermore, they reported that Safranal can situate between α and β tubulin through a hydrogen bond with Gly 142 and some hydrophobic interactions. They hypothesized that a decline of tubulin assembly could result from tubulin structural changes through safranal bindings [9]. Furthermore, treating hepatocarcinoma cells with Safranal caused DNA double-strand breakage; induce apoptosis and cell death [26].

In addition, generating ROS molecules by Fe_3_O_4_ NPs could also damage cell components, especially DNA molecules and cytoplasmic membrane as well as mitochondrial membrane. Khan et al. associated the oxidative stress generated with iron oxide nanoparticles with the anticancer effects on lung epithelial cancer cells [27]. Measuring the ROS level in control and nanoparticle-treated cells indicated a significantly higher level of ROS molecules in the treated cells that is in agreement with previous reports. Therefore, the cytotoxic effect of Fe_3_O_4_@Glu-Safranal NPs could be mainly associated with the inhibitory effect of Safranal on cancer cells as well as the oxidative stress generated by iron oxide NPs.

Comparing the cytotoxic effect of Fe_3_O_4_@Glu-Safranal NPs on liver cancer cells and normal fibroblast cells revealed that the nanoparticles were significantly more toxic for cancer cells. The higher membrane permeability and nutrient uptake with cancer cells seem to be responsible for their higher susceptibility to the nanoparticles. Since the nanoparticles are coated with glucose and considering that glucose is an essential nutrient for the growth of human cells, the presence of glucose facilitates the entry of nanoparticles into the cells. On the other hand, due to the higher rate of reproduction and metabolic activities of cancer cells compared to healthy cells, the nutritional requirement and uptake of these cells are naturally higher than normal cells which could lead to the higher penetration of nanoparticles into cancer cells [28]. This hypothesis explains the higher sensitivity of cancer cells compared to normal cells; however, further investigations are required.

In this study, glucose was used to coat iron oxide nanoparticles. Coating metal nanoparticles with glucose have several advantages. Glucose can act as an intermediate molecule to facilitate the conjugation of Safranal to iron oxide nanoparticles. Coating with glucose increases the biocompatibility of the nanoparticles and, as a result, may reduce their side effects. Since glucose is an essential nutrient for human cells, and cancer cells have a naturally high nutritional requirement, coating with glucose may facilitate the entry of nanoparticles into cancer cells and can increase their anticancer effectiveness.

Flow cytometry analysis of the control and nanoparticles treated cells revealed that treating with Fe_3_O_4_@Glu-Safranal NPs considerably increased the population of necrotic and apoptotic cells. Cell necrosis and apoptosis are two common outcomes of damage to cell components. As described above, Safranal and iron oxide could exert several cytotoxic effects on cancer cells that could inhibit DNA replication and cell proliferation. As observed by cell cycle analysis, treating of liver cancer cells with nanoparticles caused higher cell cycle arrest at the G2/M phase. Similar to our finding, Zhang et al. reported that Safranal induces apoptosis and cell cycle arrest at the G2/M phase in colon carcinoma cells [29]. Also, Al-Hrout et al., found that treating hepatocellular cancer cells with Safranal, induced cell cycle arrest at the G2/M phase and cell apoptosis, which is in agreement with our results [26].

Following damage to different parts of the cell, especially DNA, mediators of pro-apoptotic pathways are activated and lead the cell to the path of programmed death. It was reported Safranal could enhance the transcription of proapoptotic genes, which can lead cancer cells into apoptosis [10, 26]. Therefore, it seems that the increase in the population of apoptotic cells is due to the damage to different parts of the cell by Fe_3_O_4_@Glu-Safranal NPs and subsequent activation of proapoptotic pathways. To elucidate this, it would be helpful to conduct molecular studies on the level of expression and activity of apoptotic pathway proteins in Fe_3_O_4_@Glu-Safranal NPs treated cells.

## Conclusion

In this work, the anticancer effect of Fe_3_O_4_@Glu-Safranal NPs liver cancer cells was characterized. Our results revealed that Fe_3_O_4_@Glu-Safranal is an efficient antiproliferative agent against cancer cells that could induce anticancer effects through cell cycle arrest and apoptosis induction. Due to the biocompatibility of the constituent compounds of these nanoparticles, their magnetic properties, and their inhibitory effect on cancer cells, Fe_3_O_4_@Glu-Safranal NPs can be considered for further in vitro and in vivo trials against liver cancer.

## Data Availability

The data produced in this research are ready by the corresponding author on request.

## Declarations

### Approval for human experiments

It is not applicable.
